# Comparison of the Photosynthetic Yield of Cyanobacteria and Green Algae: Different Methods Give Different Answers

**DOI:** 10.1371/journal.pone.0139061

**Published:** 2015-09-22

**Authors:** R. Milou Schuurmans, Pascal van Alphen, J. Merijn Schuurmans, Hans C. P. Matthijs, Klaas J. Hellingwerf

**Affiliations:** 1 Molecular Microbial Physiology Group, Swammerdam Institute for Life Sciences, University of Amsterdam, Amsterdam, The Netherlands; 2 Aquatic Microbiology, Institute for Biodiversity and Ecosystem Dynamics, University of Amsterdam, Amsterdam, The Netherlands; 3 Photanol BV, Amsterdam, The Netherlands; CEA-Saclay, FRANCE

## Abstract

The societal importance of renewable carbon-based commodities and energy carriers has elicited a particular interest for high performance phototrophic microorganisms. Selection of optimal strains is often based on direct comparison under laboratory conditions of maximal growth rate or additional valued features such as lipid content. Instead of reporting growth rate in culture, estimation of photosynthetic efficiency (quantum yield of PSII) by pulse-amplitude modulated (PAM) fluorimetry is an often applied alternative method. Here we compared the quantum yield of PSII and the photonic yield on biomass for the green alga *Chlorella sorokiniana* 211-8K and the cyanobacterium *Synechocystis* sp. PCC 6803. Our data demonstrate that the PAM technique inherently underestimates the photosynthetic efficiency of cyanobacteria by rendering a high F_0_ and a low F_M_, specifically after the commonly practiced dark pre-incubation before a yield measurement. Yet when comparing the calculated biomass yield on light in continuous culture experiments, we obtained nearly equal values for both species. Using mutants of *Synechocystis* sp. PCC 6803, we analyzed the factors that compromise its PAM-based quantum yield measurements. We will discuss the role of dark respiratory activity, fluorescence emission from the phycobilisomes, and the Mehler-like reaction. Based on the above observations we recommend that PAM measurements in cyanobacteria are interpreted only qualitatively.

## Introduction

A wide range of sustainability applications underline the important role of oxyphototrophic microorganisms (in particular cyanobacteria and green algae) in today’s research in biotechnology and synthetic biology [1,2]. For such applications, organisms are desired that convert solar energy into chemical free energy with the highest possible efficiency. In case of direct application of solar energy for algal culturing, a range of variables need to be taken into account, such as temperature, gas exchange, algal density, layer thickness and mixing regimes [3,4]. Also the solar lighting as such is highly variable in several ways: sinusoidal day/night rhythms, plus superimposed changes in light intensity due to cloudy skies and (self-) shading [5]. Combined, all these variables present a large technological challenge to determine the phototropic growth yield on light under relevant conditions. In most ‘algal’ biotechnology literature, biomass yields on light are described as biomass per total cumulated daily light dose [3,6,7]. However, optimal exploitation of an organisms’ growth potential relies very much on how the inherent properties of bioreactors can be adjusted to accommodate it optimally. A commonly used alternative approach for determination of the growth efficiency on light is the pulse amplitude modulated fluorimetry technique, which estimates photosystem II (PSII) quantum yield [5,8–10]. In reviewing a range of publications, quite different apparent quantum yield values emerge when comparing plants (as high as 0.8 [11–13]), green algae (around 0.7 [14,15]), and cyanobacteria (around 0.4 [16,17]). These quite large differences in apparent quantum yield between clades of oxygenic phototrophs have previously been studied and the low values in cyanobacteria have been attributed mainly to interfering fluorescence emitted by the phycobilisome light harvesting antennae [18–20]. Accordingly, we argue that PSII quantum yield values as such are not a correct measure for comparison of the overall photosynthetic efficiency of different oxygenic phototrophs. Indeed, cyanobacteria risk to be incorrectly marked as less efficient [21]. Despite of this, usage of the PAM method may serve algal and cyanobacterial mass culture management very well, provided that data are used for qualitative comparison of growth performance for each single strain individually.

With that restraint, detailed analyses of the PAM signal (and its dynamics) can be used as a qualitative reporter technique for a host of physiological characteristics of chlorophyll-based oxygenic photosynthesis. Examples are: the level of photochemical and non-photochemical quenching [22], the rates of linear and cyclic electron transfer around PSI [23,24] and the maximal efficiency of photochemistry/charge separation in PSII, referred to as the quantum yield of PSII; ϕ_PSII_ [25]. A generally accepted protocol for PAM measurements has been established, in combination with an associated nomenclature [26]. Here the variable fluorescence of PSII is determined via comparison of the minimal fluorescence after dark incubation (F_0_), reflecting a state in which all PSII centers are open, the maximal fluorescence as observed when PSII is saturated with an intense pulse of light (F_M_), reflecting a state in which all PSII centers are closed, and the modulated fluorescence signal in the presence of actinic light (F) which ranges in between both limits.

For chloroplasts from plants and green alga the assumptions inherent to this technique are generally accepted, and have been widely applied [9,22,27,28]. However, in cyanobacteria similar straightforward measurement of the signals originating from variable PSII-derived fluorescence is hampered by the presence of: 1) interfering non-variable background fluorescence from the specific phycobilisome antenna systems of cyanobacteria [19,20]; 2) respiratory electron flow that overlaps with the photosynthetic electron flow in the thylakoid membrane, generating a more reduced PQ-pool in the dark as compared to plants and algal chloroplasts [29–31]; and 3) a substantially higher PSI/PSII expression ratio, resulting in an increased contribution of non-variable PSI fluorescence to the dark F_0_ fluorescence level [18,20]. Although at wavelengths shorter than 700 nm the PSI contribution is negligible, for λ > 700 nm it contributes between 30 and 50% of the total fluorescence emission (F_0_) in C3- and C4–plants, respectively [32,33]. In PAM measurements, the chl *a* fluorescence signal is recorded with cut-off filters that allow light to pass with λ > 696 nm and by consequence a higher PSI/PSII ratio intrinsically raises the level of non-variable fluorescence. Hence, using the standard data interpretation and calculus procedures, a lower apparent PSII quantum yield will be attributed to cyanobacteria.

Measuring the rate of oxygen evolution also provides an indication of how well PSII is functioning and how many electrons are being released into the Z-scheme at a particular light intensity. Such measurements are often conducted using a Clark-type oxygen electrode or an optode but this does not provide information on simultaneous oxygen production and consumption [17,34]. In attempts to overcome this limitation it is often assumed that the rate of respiration that is measured in the dark will not be exceeded by the rate of oxygen consumption in the light, or even that this rate of respiration will stay constant, independent of the light intensity. Oxygen evolution rates measured in the light are therefore often ‘corrected’, via addition of the rate of oxygen consumption that is measured in the dark [35,36]. However, previous studies have already shown that oxygen consumption in the light can inhibit respiratory electron flow under low light conditions [37,38] and under moderate to high light conditions oxygen consumption extends to much above the dark rate [39–41].

In the present work we elaborate on two analysis techniques used to estimate the relative efficiency of oxygenic phototropic growth on light; PAM based PSII yield estimation and oxygen exchange. The data presented demonstrate that the absolute PAM derived PSII yield does not permit direct comparison between different phototrophic taxa. The mechanistic reasons behind the aberrantly low photosynthetic yield estimation by PAM in cyanobacteria or the low oxygen production in high light have been analyzed using *Synechocystis* sp. PCC 6803 (*Synechocystis*) mutants deficient in the respiratory terminal oxidases, the main NADPH dehydrogenase, the Mehler-like flavodiiron proteins and the phycobilisome light harvesting antenna. The results clarify why the convenient instrumental PAM and oxygen optode analysis techniques renders very different insight into growth efficiency for the species compared, whereas the actual measurement of growth efficiency on light in continuous culture results in very similar values for biomass conversion between *Synechocystis* and *Chlorella sorokiniana* 211-8K (*Chlorella*). It is concluded that the PAM technique cannot be used for direct comparison between different clades of oxygenic phototrophs.

## Results

**Fig 1:**
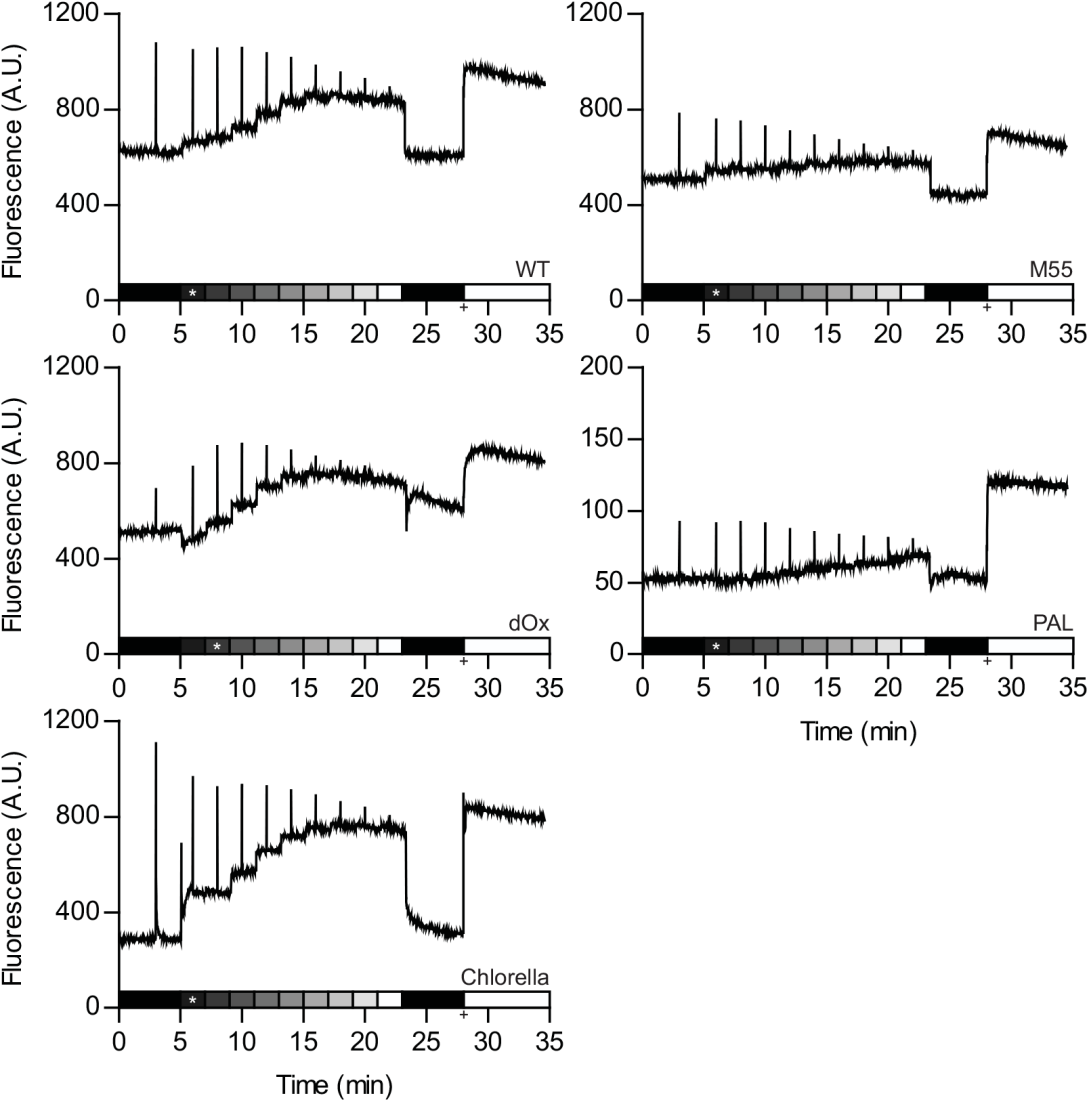
Variable chl *a* fluorescence as measured with a pulse-amplitude modulated (PAM) fluorescence in wild type *Synechocystis*, three of its mutant derivatives, and the green alga *Chlorella*. Batch cultures of wild type *Synechocystis*, the *ndhB* deletion mutant M55, the triple terminal oxidase deletion mutant ΔOx, the phycobilisome-free PAL mutant and the green alga *Chlorella* were grown in blue/red fluorescent light (see Materials and Methods). Cells for chl *a* fluorescence recordings were harvested in the linear phase of growth and incubated in a flat-panel flask. Prior to the experiment the cultures were dark adapted for 30 minutes and exposed to 2 minute illumination periods with red (659 nm) light with increasing light intensity ranging from 30–400 μmol photons m^-2^ s^-1^, as indicated by the shaded bar. Light intensities used were 30, 60 and 100 μmol photons m^-2^ s^-1^ followed by 50 μmol photons m^-2^ s^-1^ increases at each step until 400 μmol photons m^-2^ s^-1^. The asterisk indicates the growth light intensity of the pre-culture. In the middle of each period (i.e. after 1 min) the cells were subjected to a strong ‘white’ light pulse (2000 μmol photons m^-2^ s^-1^). Following the actinic light series, the cells were left in darkness for 5 minutes. The + marks when DCMU was added at a final concentration of 20 μM together with strong red light at an intensity of 400 μmol photons m^-2^ s^-1^.

Chl *a* fluorescence traces were recorded with a PAM fluorimeter from batch cultures in the linear phase of growth (i.e. the light limited phase) of *Synechocystis*, its various mutants, and *Chlorella* (see Materials and Methods and S1 Fig for more details), as shown in Fig 1. The PSII quantum yield was calculated as ϕPSII = (F_M_-F_0_)/F_M_, variable fluorescence as F_V_ = F_M_-F_0_, photochemical quenching (q_P_) as q_P_ = (F_M_'-F)/ (F_M_'-F_0_') and the non-photochemical quenching (q_N_) as q_N_ = 1-((F_M_'-F_0_')/ (F_M_-F_0_)) [26]. For F_0_ the chl *a* fluorescence emission intensity after dark adaptation was used and for F_0_' the lowest intensity after illumination. For F_M_, the highest value after a saturation pulse in the dark or after addition of DCMU was used.

The minimal fluorescence recorded in this study is reached for *Chlorella* and for the PAL mutant (*Synechocystis* without phycobilisomes) after dark adaptation (F_0_). Interestingly, for WT *Synechocystis* and the NADPH dehydrogenase deletion mutant M55, the lowest value of fluorescence emission was recorded after the actinic light application (F_0_') was completed, and in the terminal respiratory oxidase deletion mutant ΔOx it is recorded at the onset of the actinic illumination (Fig 1, Table 1). Additionally, the F_M_ value of ΔOx was lower after dark adaptation than when measured at an actinic light intensity comparable to the growth light intensity (* in Fig 1). The other strains do not display this trend.

**Table 1:** The respiratory chain and the phycobilisomes affect chl *a* parameters.

	chl *a*	F_0_	F_0_'	F_M_	F_V_	ϕ_PSII_ max	ϕ_PSII_ GL
**WT**	2.95 ± 0.025	601 ± 2	585 ± 1	1098 ± 17	474 ± 15	0.45 ± 0.006	0.37 ± 0.004
**M55**	1.63 ± 0.005	471 ± 18	402 ± 14	740 ± 45	357 ± 12	0.36 ± 0.015	0.27 ± 0.014
**ΔOx**	2.71 ± 0.046	494 ± 9	494 ± 3	883 ± 2*	356 ± 10*	0.44 ± 0.008*	0.36 ± 0.014
**PAL**	3.18 ± 0.009	49 ± 1	46 ± 1	129 ± 3*	80 ± 3*	0.62 ± 0.014*	0.46 ± 0.016
**Chlorella**	3.61 ± 0.093	255 ± 20	277 ± 19	1045 ± 66	780 ± 47	0.76 ± 0.004	0.49 ± 0.008

Values shown were derived from the experiments as depicted and described in Fig 1. All values were normalized to OD_730_. chl *a*, chlorophyll *a* concentration in mg L^-1^; F_0_, level of fluorescence in the dark; F_M_, fluorescence measured by applying a strong light pulse (2,000 μmol photons m^-2^ s^-1^) in the dark; F_V_, variable fluorescence (F_M_-F_0_); ϕ_PSII_, quantum yield of PSII ((F_M_-F_0_)/F_M_) calculated using F_0_ and F_M_ (max) or F_0_' and F_M_' under growth light conditions (GL).

*, derived using F_M_ values after addition of 20 μM DCMU in the light (400 μmol photons m^-2^ s^-1^). Values are averages of duplicate measurements with standard deviation.

The PSII yield values derived from data acquired from dark-adapted cells differ widely between *Synechocystis*, its mutants, and *Chlorella*. The highest yield values were recorded for *Chlorella* and the PAL mutant of *Synechocystis*, even though the PAL mutant has a much lower total fluorescence signal and a very small variable fluorescence (F_V_). Markedly lower yield values were obtained for *Synechocystis* WT and the respiratory M55 and ΔOx mutants (Table 1). However, when comparing the data acquired in the presence of actinic illumination of an intensity comparable to growth light intensity, the differences are much smaller (Table 1).

**Fig 2:**
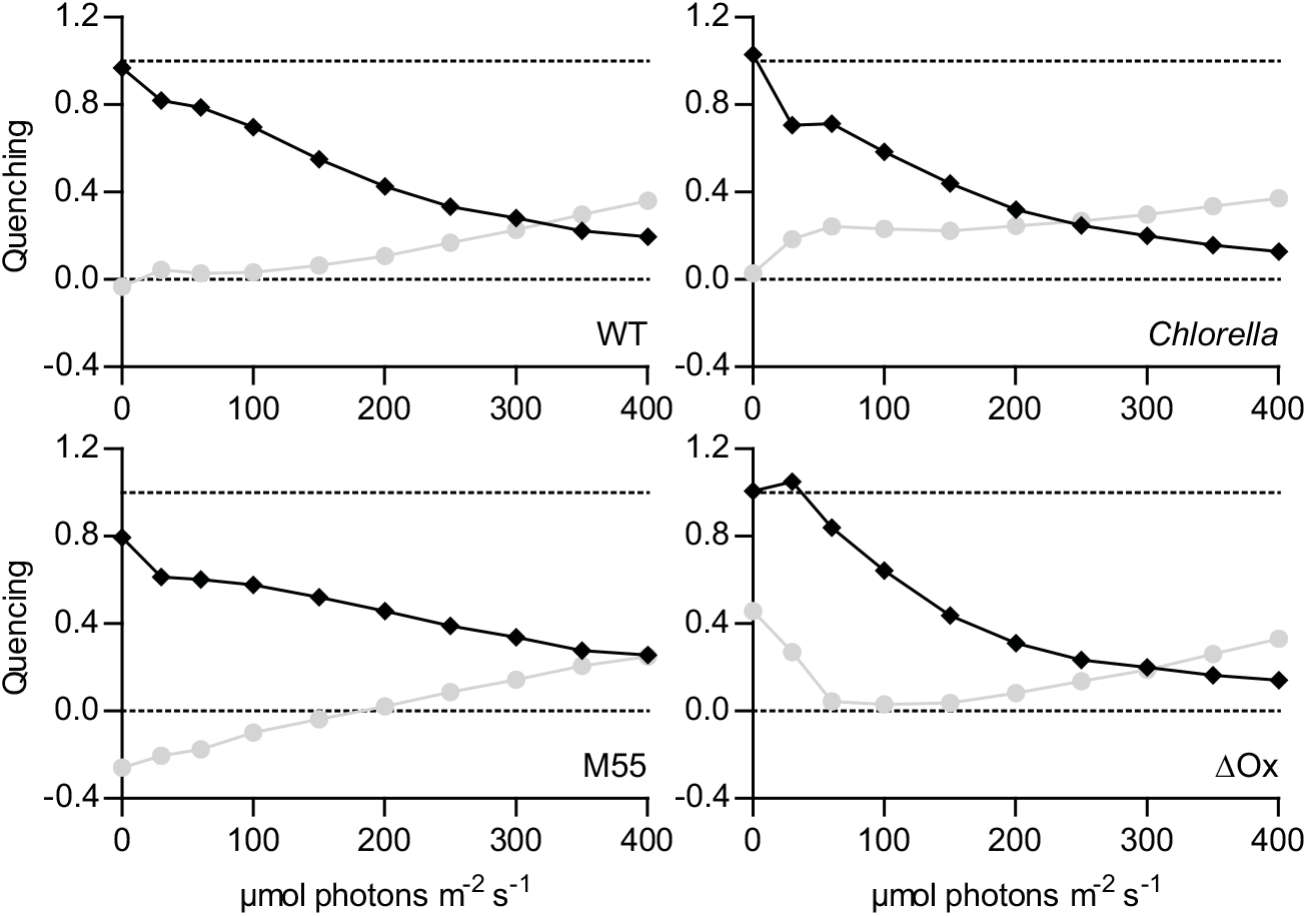
Light-intensity dependence of the photochemical and non-photochemical quenching in wild type *Synechocystis*, its mutant derivatives M55 and ΔOx, and the green alga *Chlorella*. Light-intensity dependent levels of photochemical (♦, q_P_) and non-photochemical quenching (●, q_N_). Values were derived from the data described and depicted in Fig 1. Q_P_ was calculated as (F_M_'-F)/(F_M_'-F_0_') and q_N_ as 1-((F_M_'-F_0_')/(F_M_-F_0_)) with: F_0_, lowest level of fluorescence after dark adaptation; F_0_' lowest level of fluorescence after light incubation; F, steady-state fluorescence in the presence of actinic light; F_M_, fluorescence upon a strong light pulse on dark adapted cells or after addition of DCMU (ΔOx mutant only). F_M_', fluorescence upon a saturation pulse in the presence of actinic light. Data are the average from duplicate measurements, standard deviation was between 1 and 5%.

At the addition of increasing amounts of actinic light, the photochemical quenching (q_P_, Fig 2) declines more sharply in *Chlorella* at low actinic light intensities than it does in the *Synechocystis* strains. This is also directly visible in Fig 1: the increase in fluorescence when the light is first switched on is much stronger for *Chlorella* than it is in *Synechocystis*. A similar trend is observed for non-photochemical quenching (q_N_, Fig 2); a big increase for *Chlorella* and a much more modest change for *Synechocystis*. In the absence of the terminal oxidases, the chl *a* fluorescence response of *Synechocystis* to increasing intensities of actinic illumination rises much more steeply (Fig 1), and is accompanied by a more steep decrease in q_P_ and increase in q_N_ (Fig 2, ΔOx). Without the NDH-1 complex, the chl *a* fluorescence response of *Synechocystis* becomes even more gradual than in the WT (Figs 1 and 2, M55). Because of the small dynamic range in the signal recorded from the PAL mutant and the large difference between light and DCMU derived signals, the PAL mutant was deemed to have unreliable values for F_M_' and was not included in Fig 2.

Next, the photosynthetic efficiency of growth of *Chlorella* and two of the *Synechocystis* strains (WT and PAL mutant) was determined in continuous culture by determining the number of absorbed photons for the production of 1 g of biomass. The two wild type organisms, i.e. *Synechocystis* and *Chlorella*, were grown in a flat panel chemostat [42] at a fixed growth rate (about 0.07 h^-1^) as set by the dilution rate. We chose to use the same wavelength (659 nm) both for growth in the chemostat and for actinic illumination in the PAM experiments. By determining the amount of light absorbed by the culture and its biomass content in steady state, the photosynthetic efficiency was calculated, expressed as the number of photons absorbed per gram of dry weight formed. The two strains were grown with nitrate as their nitrogen source. Because nitrate assimilation requires a considerable amount of reducing equivalents and cyanobacteria tend to contain a higher percentage of nitrogen than green algae, we also calculated the photon yield for generating electron pairs for CO_2_ fixation and nitrate assimilation. For this we used the elemental compositions from three published studies per strain. The data presented in Table 2 indicate that *Chlorella*, with the significantly higher PSII quantum yield according to the PAM measurements, has a similar photonic yield on biomass and a slightly lower yield on electron pair generation than *Synechocystis*.

**Table 2:** Comparison of the efficiency of photosynthesis in a pro- and a eukaryotic oxyphototroph in terms of moles of photons required for biomass production and electron pair generation at moderate light intensities.

Species	I_in_	I_out_	OD_730_	g DW L^-1^	μ h^-1^	mol hν g DW^-1^	hν/ 2e^-^
*Synechocystis* WT	76.3	24 ± 0.8	0.49 ± 0.01	0.091 ± 0.004	0.071	0.58 ± 0.019	4.9 ± 0.23
*Chlorella*	65.9	21.4 ± 0.7	0.59 ± 0.01	0.080 ± 0.005	0.070	0.57 ± 0.027	6.0 ± 0.49
*Synechocystis* PAL	71.1	17.1 ± 1.5	0.65 ± 0.05	0.161 ± 0.006	0.023	1.04 ± 0.038	n.a.

Biomass yield on photons for *Synechocystis* WT, *Chlorella* and *Synechocystis* PAL. Cells were growing in red (659 nm) light or red and blue (447 nm) light. I_in_, light intensity in μmol m^-2^ s^-1^ passing through the culture vessel containing only medium; I_out_, light intensity in μmol m^-2^ s^-1^ passing through the culture vessel containing cell culture; OD_730_, optical density at 730 nm; g DW L^-1^, biomass density in gram dry weight per liter; μ h^-1^, growth rate per hour; mol hν/g DW, growth yield in mol photons absorbed per g biomass. hν/2e-, # of photons absorbed per electron pair for nitrogen and carbon incorporation. The elemental composition of the cells was taken from literature: for *Synechocystis* [43–45] and for *Chlorella* [46–48] and averaged. The values shown are averages of three measurements with standard deviation.

The PAL mutant, which has a much higher apparent PSII quantum yield than WT *Synechocystis*, as indicated by the PAM measurements (Table 1), was subjected to the same analysis. However, as this mutant does not, or only very poorly, grow with red light only, we had to add blue light to achieve an acceptable growth rate (> 0.02 h^-1^; see Table 2 and Materials and Methods). Under these assay conditions the PAL mutant of *Synechocystis* turned out to be much less efficient than the wild type *Synechocystis* grown in moderate red light intensities: around 1 mole photons were needed by the PAL mutant to produce 1 gram of biomass versus 0.58 mol photons for the WT (Table 2).

**Fig 3:**
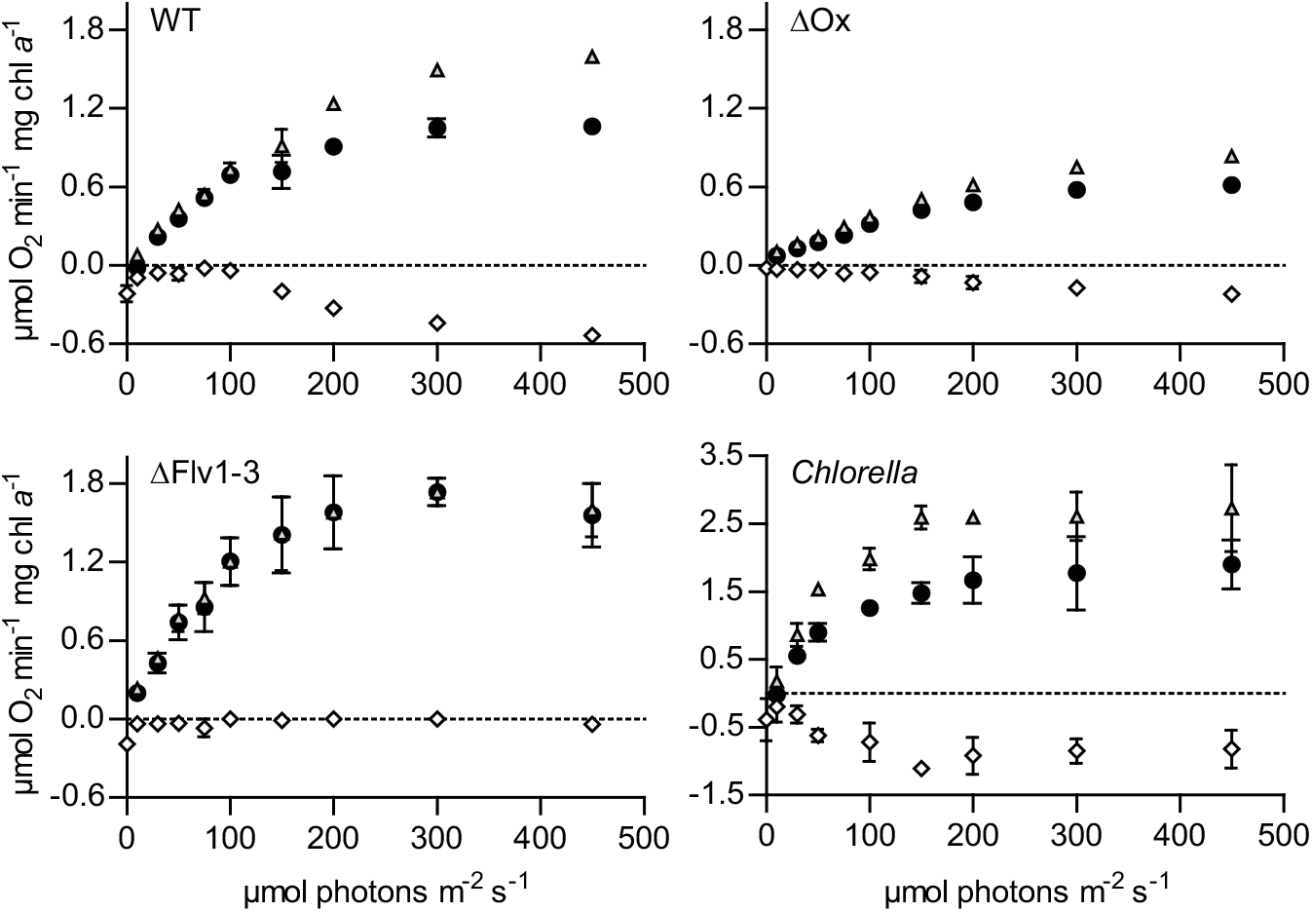
Light-intensity dependence of net oxygen production and consumption, as measured with a membrane-inlet mass spectrometer (MIMS), of wild type *Synechocystis*, its ΔOx and ΔFlv1-3 mutant derivatives and *Chlorella*. Oxygen exchange was measured with MIMS as a function of increasing red light (659 nm) intensity in wild type *Synechocystis* (WT), the terminal-oxidase deletion mutant (ΔOx), the Mehler-like reaction deletion mutant (Δflv1-3) and *Chlorella sorokiniana* (*Chlorella*). Cells were grown in batch with 30 μmol photons m^-2^ s^-1^ blue/red fluorescent light (see Materials and Methods) in BG-11 medium complemented with 25 mM NaHCO_3_. Prior to measurements, the cells were washed once and re-suspended in fresh BG-11 medium with 50 mM NaHCO_3_. Cells were dark adapted for 30 minutes prior to the experiment. Experiments with *Synechocystis* and its mutant derivatives were conducted at 18°C and *Chlorella* at 30°C. ●, oxygen evolution measured as an increase in the concentration of ^32^O_2_; ◊, oxygen uptake measured as a decrease in the concentration of ^36^O_2_; Δ, net rate of photosynthesis (i.e. rate of net oxygen evolution + rate of oxygen uptake). Values shown are the average of duplicate measurements with standard deviation.

Membrane-inlet mass spectrometry (MIMS) was used to determine quantitatively the extent of interference of oxygen consumption with the net rate of light-dependent oxygen evolution. We analyzed oxygen uptake and evolution rates in *Chlorella*, wild type *Synechocystis*, in the triple oxidase mutant ΔOx, and in a mutant deficient in the Mehler-like reaction (Δflv1-3; see Materials and Methods for details). Fig 3 shows that in *Synechocystis* under very low actinic light conditions there is a low rate of oxygen uptake via the respiratory oxidases, and that this rate is much lower than dark respiration which is 0.22 and 0.19 μmol O_2_ min^-1^ mg chl *a* ^-1^ in the WT and the Δflv1-3 mutant, respectively. Furthermore, in the presence of functional oxidases, dark respiration is fully inhibited already at very low light intensities (i.e. 10 to 20 μmol photons m^-2^ s^-1^, Fig 3). Starting at around 150 μmol photons m^-2^ s^-1^, the oxygen uptake rate starts to exceed the rate of dark respiration. This coincides with the point where the increase in the rate of ^32^O_2_ evolution changes from a linear increase to saturation behavior with respect to light intensity (Fig 3). In the ΔOx mutant the increase in oxygen uptake rate shows a trend similar to the WT, while in the Δflv1-3 mutant oxygen uptake during illumination is fully abolished (Fig 3). In *Chlorella*, oxygen uptake is only lower than dark respiration at the lowest light intensity; with increasing light intensity oxygen uptake increases and stabilizes around 150 μmol photons m^-2^ s^-1^, which is at a higher light intensity than the point where ^32^O_2_ evolution starts to show saturation behavior (around 100 μmol photons m^-2^ s^-1^). Due to circumstance the oxygen evolution/consumption rates of *Chlorella* were measured in a slightly different set-up and a different temperature (30 vs 18°C) than those of *Synechocystis* and its mutant derivatives. As a control *Synechocystis* WT was also tested in the alternative set-up. The shape of the curve at 30°C was similar to the ones recorded at 18°C, and the maximal net rate of photosynthesis of *Synechocystis* WT at 30°C was 7.8 ± 0.3 μmol O_2_ min^-1^ mg chl a^-1^.

## Discussion

Not all photons harvested by the photosynthetic pigment complexes are productively used for NADPH and ATP synthesis via electron transfer by the components of the Z-scheme. Excess light energy can be non-photochemically quenched via specific dissipation reactions and released as heat. Based on well-established knowledge, largely acquired in plant photosynthesis research, a non-invasive method was developed for monitoring such processes [22]. The PAM technique permits us to distinguish which portion of the light that PSII receives is used productively for linear photosynthetic electron flow (q_P_), and which portion is lost non-photochemically (q_N_). Most protocols for the use of the PAM technique include a dark pre-incubation of the cells to determine the minimal (F_0_) and maximal fluorescence (F_M_) emission, from which the amount of energy available for linear electron flow can be calculated as the maximal quantum yield of PSII [25,49]. The underlying assumption is that in darkness the primary quinone of PSII (Q_A_) slowly oxidizes, which in turn triggers photosynthetic antennae to associate with PSII to not only result in a low fluorescence dark state, identified as F_0_, but also to provide a full antenna complement for PSII (state I) so that maximal fluorescence (F_M_) can be derived from PSII upon exposure to an intense short flash of light.

**Fig 4:**
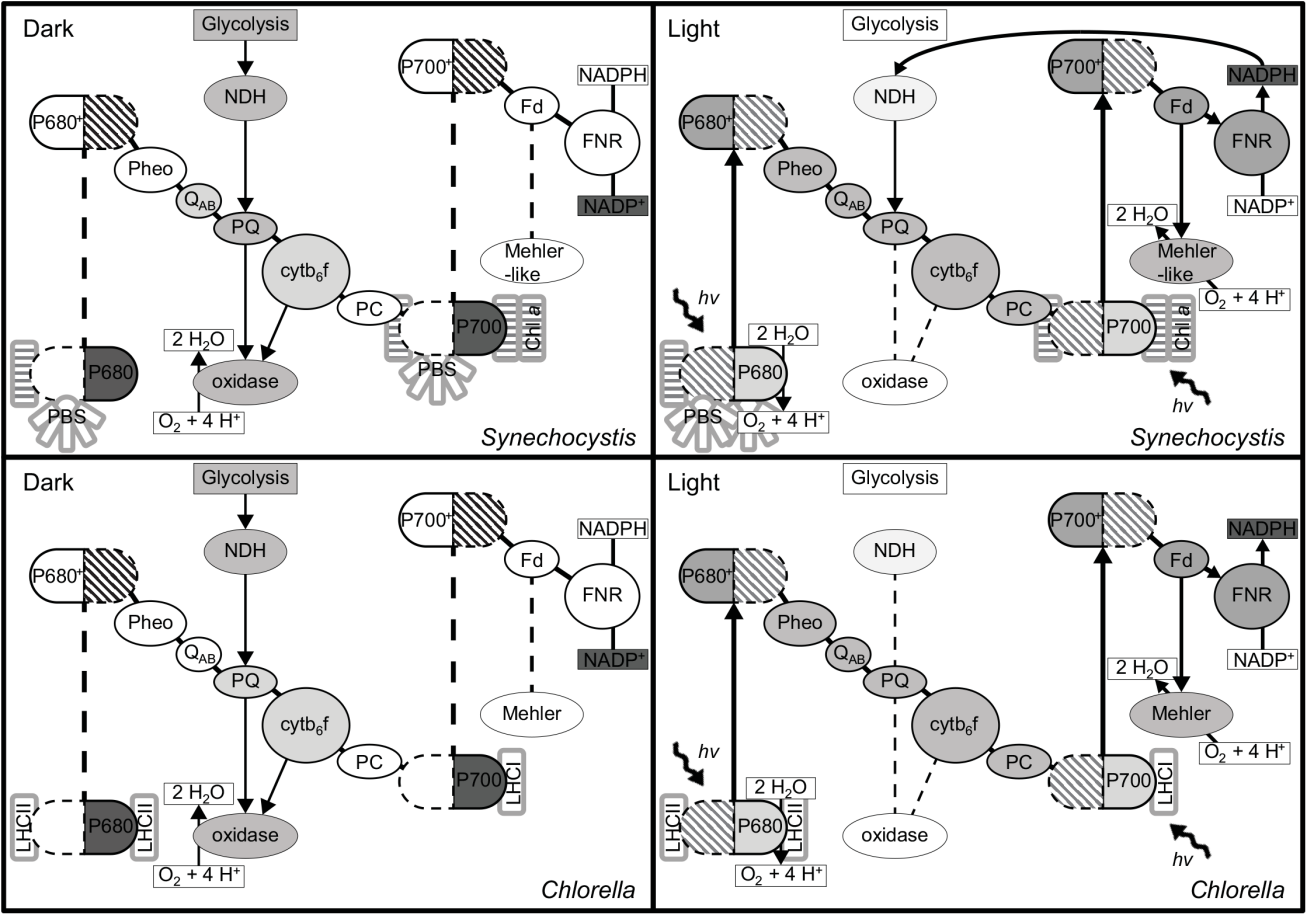
Schematic overview of the redox state of the components of the photosynthetic electron transport chain. Schematic overview of the redox state of the photosynthetic electron chain in dark (left) and light (right) for *Synechocystis* (top) and *Chlorella* (bottom). The grey color indicates the level of reduction (the darker the more reduced) of the different intermediates or, in case of P680, P700 and NADP(H), the predominant species.

However, these assumptions are incorrect with respect to cyanobacteria, where respiratory pathways intersect with photosynthetic electron flow in the same thylakoid membranes and both flows of electrons share the plastoquinone pool [29]. Through the moderating actions of respiratory dehydrogenase enzymes for electron input, and the terminal oxidases for electron efflux (from the plastoquinone pool), the photosynthetic electron transport chain, and the Q_A_ of PSII does not become fully oxidized in the dark (see also [31] and Fig 4), which leads to a higher apparent F_0_ as attested by the lower F_0_' values for *Synechocystis* WT and M55 after actinic illumination. Fig 1 demonstrates the ability of the respiratory chain to moderate the redox potential of the PQ-pool with a more gradual increase in fluorescence (F) with increasing light intensity for the M55 strain and a more intense increase in the ΔOx strain. Fig 2 also shows some unlikely and even non-physical values for photochemical and non-photochemical quenching with the standard data evaluation protocol (see above): In the ΔOx strain, at low light intensities q_P_ is > 1.0 and for the M55 mutant the calculated q_N_ is negative up to 200 μmol photons m^-2^ s^-1^ (Fig 2). These apparent artifacts are caused by the fact that F_0_' is lower than F_0_ in the M55 strain, and in the case of the ΔOx strain, that F_M_ in the dark is not yielding the maximal level of fluorescence emission. Lower levels of F_M_ compared to F_M_' at low light intensities in cyanobacteria are not uncommon because cyanobacteria are believed to be in state II in the dark [50–52] with antenna association to PSI and not PSII. This small antenna state of PSII often results in lower levels of F_M_ fluorescence emission upon a strong light pulse [53]. However in *Synechocystis* PCC 6803 this phenomenon is not always visible and it has even been proposed that *Synechocystis* is an exeption and does not enter state II in the dark at all [19]. The fact that this phenomenon occurs in the ΔOx strain may be an argument against calling *Synechocystis* an exeption, but it may explain why it is not visible in the WT and M55 strains, possibly caused by a less stringent state II due to a less reduced PQ-pool. Campbell et al. [19] advises the use of DCMU for F_M_ determination in cyanobacteria. The electron transfer inhibitor DCMU prevents oxidation of Q_A_^-^ by Q_B_, resulting in fully reduced Q_A_ in the light, and therefore in maximal chl *a* fluorescence. Nevertheless, we observed that the fluorescence emission intensity is not always highest in the presence of DCMU (Fig 1). This may be caused by the high intensity of the actinic light used, that in combination with DCMU, may cause accelerated photo-bleaching and lower levels of fluorescence [54].

Additionally, an increased contribution of PSI to the measured (broad-band) fluorescence emission, due to higher numbers of PSI reaction centers and increased excitation due to antenna association with PSI [20] increases the F_0_ value even further. Considering all of this, it is evident that cyanobacteria emit higher levels of F_0_ and lower levels of F_M_ fluorescence with standard dark adaptation protocols than green algae and plants. When the quantum yield of PSII is calculated as (F_M_-F_0_)/F_M_ then the absolute values of F_0_ and F_M_ matter and higher levels of overall fluorescence emission will give rise to lower calculated PSII quantum yields. A clear example of this is shown in Table 1 where the *Synechocystis* strain with the smallest variable fluorescence (F_M_-F_0_) has the highest PSII quantum yield (i.e. the PAL mutant). The high yield values of the PAL mutant have already been described in previous work and the PAL mutant has been considered as a model for interpretation of PSII fluorescence data in cyanobacteria without the interference of the phycobilisomes background fluorescence [55]. Also, it has been reported that the PAL mutant compensates for the loss of phycobilisomes by increasing its PSII content [56].

In the presence of increasing actinic light intensities Q_A_ becomes more and more reduced, which increases the basal level of fluorescence (F) and lowers the apparent quantum yield of PSII. In addition, non-photochemical quenching further reduces the rate of exciton flow into PSII [57] and lowers q_P_. Subtraction of these two losses from the highest quantum yield in dark adapted cells results in the ‘active PSII quantum yield’. At growth light intensity q_N_ is very low; in cyanobacteria it is generally even at its lowest point and this information can be used to derive to which light condition the cells are adapted [19]. In Fig 2 only the ΔOx strain follows this behavior, probably caused by the lack of a visible dark state II in the other strains causing q_N_ to be lowest in the dark adapted state. Also under growth light conditions the active PSII yield or q_P_ give a good measure for oxygen evolution and CO_2_ fixation rates [19], this is then distorted by q_N_ at higher light intensities. Even though *Chlorella* maintains a higher active quantum yield under growth-light conditions than *Synechocystis* (ϕ_PSII_ GL in Table 1), the difference is much smaller than the difference between their maximal quantum yield in the dark; the q_P_ value at growth light intensities is even lowest for the green alga and the value for q_N_ is the highest at growth light intensity (Fig 2). Although we must note that these experiments were conducted in red light only, which may bypass one of the major exciton-energy dissipating mechanisms in cyanobacteria and thus possibly underestimate the level of q_N_ that may occur in white light. This mechanism is referred to as exciton quenching by the orange carotenoid protein (OCP) and is activated by strong blue light [58]. Nonetheless, direct comparison of the performance of both species as measured with the PAM technique gives much closer values if the yield is determined in the presence of actinic light. In fact, when comparing the PAL mutant of *Synechocystis* with *Chlorella*, the PSII quantum yield is almost equal (Table 1). This implies that one could attribute the lower apparent quantum yield of PSII in *Synechocystis* WT predominantly to PBS background fluorescence. Recently Acuña et al [52] have modelled the chl *a* fluorescence signal in cyanobacteria and found evidence that suggests that without the PBS component the PSII quantum yield values for green algae and cyanobacteria are indeed equal. In the future such a model could be used to extract the different components that make up the PAM signal, to provide reliable information on PSII function and the influence of various non-photochemical quenching mechanisms. However currently the model described by Acuña et al [52] only includes a parameter for OCP which was most likely not activated in the experiments described here.

Use of an alternative method for photosynthetic yield determination, i.e. measurement of the number of photons required to produce biomass or to generate electron pairs from water, also leads to the conclusion that *Chlorella* and *Synechocystis* have a similar efficiency in light-energy conversion when growing at approximately the same growth rate in steady state continuous culture (Table 2). *Chlorella* and *Synechocystis* have a somewhat different biomass composition, especially when it comes to their nitrogen content. Although both strains contain approximately 50% of biomass in carbon, *Chlorella* contains only around 6.5% nitrogen when grown under replete conditions [46–48] while *Synechocystis* contains around 11.5% nitrogen [43–45]. The cultures in this study were grown on nitrate and CO_2_, which require 8 and 4 electrons for incorporation into biomass, respectively. When taking this difference in biomass composition into account, *Synechocystis* is slightly more efficient than *Chlorella* in the liberation of electrons from water. Consistent with this, in direct growth competition experiments cyanobacteria have generally been reported to out-compete green algae under light-limited growth conditions [59–61]. One of these studies even demonstrated that *Synechocystis* has a (slight) competitive advantage over *Chlorella vulgaris* [60]. In our experiments the PAL mutant could not be directly compared with the WT because it cannot sustain sufficiently high growth rates under conditions of moderate light intensities. However recent publications [36,62] have shown that truncation of phycobilisome antennae in cyanobacteria can improve biomass production in dense cell cultures, but only under high light conditions.

Measuring the rate of oxygen evolution also provides an indication of how well PSII is functioning and at what rate electrons are being released into the Z-scheme at a particular light intensity. The decrease in the rate of respiration at low light intensities (Fig 3) compared to the rate in the dark is known as the Kok-effect [38] and is likely caused by interference of light-driven linear electron transfer; PSI may withdraw electrons from the PQ pool more efficiently than the respiratory oxidases [63]. Back pressure of light-driven electron flow on respiratory electron flow through the common high-energy intermediate, the proton motive force, however, may play a role as well [64]. Previous studies using the MIMS technique have already indicated the Mehler-like reaction to be the main culprit in light-dependent oxygen uptake [34,65]. In this study we further confirm these results by revealing differences in the rate of oxygen consumption in the light, of mutants deficient in respiration and in the Mehler-like reaction (Fig 3). We observed that the *Synechocystis* WT consumes up to 34% of the total rate of oxygen evolution at PSII via the Mehler-like reaction at the highest light intensity (Fig 3). *Chlorella* on the other hand consumes around 36% of the total rate of oxygen evolution at all light intensities tested, exept for the lowest one (10 μmol photons m^-2^ s^-1^). Although in this setting we cannot determine which fraction of this oxygen consumption can be attributed to the true Mehler reaction, it does seem that oxygen consumption in the light has a different function or origin in green algae compared to cyanobacteria because it appears to be a constant factor, and not to be subject to light saturation. This finding does not put cyanobacteria at a perceived disadvantage of lower maximal oxygen evolution rates (P_max_), because the percentage of oxygen consumed is similar in both organisms at maximal oxygen evolution rates. The net oxygen exchange, as measured with an electrode or optode, is more indicative for growth rate and CO_2_ fixation rates and this parameter will therefore most likely be sufficient for a meaningful estimate. However it is important to keep this in mind when determining oxygen production based P_max_ values as an indicator of PSII activity and (linear) electron transport rates, because the Mehler(-like) activity, and possibly other forms of oxygen consumption will lead to an underestimation of these parameters.

We conclude that PAM signals in cyanobacteria are moderated by the respiratory electron transfer chain, leading to a dark state II, and an artificially heightened F_0_ by background fluorescence from the PBS and PSI. Together these factors cause the PAM method to grossly underestimate the photosynthetic potential of PSII of cyanobacteria. Further complications due to changing levels of spillover of PBS fluorescence and additional moderating activity of the intertwined respiratory pathway [29,66,67] and a wide array of cyclic electron flow pathways [24,68] even further complicate the matter. Nevertheless, the PAM technique can still be useful when used on a single organism to monitor relative changes in PSII yield and (non-)photochemical quenching [14,69], and perhaps to estimate other photosynthetic parameters such as O_2_ evolution and CO_2_ fixation rates, provided they are calculated at growth light intensity [19]. As long as no importance is attached to absolute values of calculated PSII efficiencies, especially from dark adapted samples, PAM signals from cyanobacteria can be very informative to quickly detect stressful conditions and monitor adaptation responses to, for instance, changing light conditions during the day.

Still, we emphasize that use of the PAM signal to compare photosynthetic yields between organisms can give rise to absolutely false predictions on light energy conversion efficiency and provides little solid information regarding the quantitative potential of cyanobacteria and algae in the upcoming bio-based economy. Determining the photonic yield of biomass formation (or CO_2_ fixation) under relevant growth conditions, will be more appropriate and informative instead.

## Materials and Methods

### Strains and culture conditions

*Synechocystis* sp. PCC 6803 was kindly provided by Devaki Bhaya, Carnegie Institution for Science, Stanford, USA. The *Synechocystis* ΔOx strain that lacks all three endogenous cytochrome oxidases [70] was provided by Wim Vermaas, Arizona State University, Arizona USA. The *ndhB* deletion mutant M55, deficient in the NDH-1 complex [71] was provided by Teruo Ogawa, Nagoya University, Chikusa, Nagoya, Japan. The Δflv1/flv3 mutant deficient in the Mehler-like reaction [72] was provided by Eva-Marie Aro, University of Turku, Turku, Finland. The PAL mutant, lacking all phycobilisome pigments [73] was provided by Ghada Ajlani, Institut de Biologie et de Technologie de Saclay, Gif-sur-Yvette, France. *Chlorella sorokiniana* 211-8k was ordered from the Göttingen culture collection.

*Chlorella sorokiniana* 211-8k, *Synechocystis* sp. PCC 6803, and its mutant derivatives, were pre-cultured in batch in BG-11 [74] medium at 30°C in a shaking incubator at 120 rpm. Growth-light was provided by Sylvania Grolux T8 fluorescent tubes (havells-sylvania.com); these tubes provide mainly blue and red light. The M55 strain was grown in the presence of 2% (v/v) CO_2_, and the other strains were grown with 25 mM sodium bicarbonate added to the medium. The ΔOx strain was grown at 60 μmol photons m^-2^ s^-1^, and all other strains were grown at 30 μmol photons m^-2^ s^-1^.

### PAM measurements

Cells were collected in the linear, light-limited, phase of growth, except for the PAL mutant which was collected in the early stationary phase, and transferred directly to a flat panel tissue culture flask (Sarstedt). The culture flask was placed in between two LED light panels (design LED 2, custom manufactured LED lamps in a cooperation between Philips Lighting Eindhoven, the Netherlands and the Technology Center of the University of Amsterdam). The optical fiber of the PAM fluorimeter was placed against the side of the vessel, perpendicular to the illuminating LED lamps; see S1 Fig for details of the set-up. PAM signals were recorded with a PAM 101 device (Walz, Effeltrich, Germany; excitation, 655 nm; emission, >696 nm). Prior to each experiment the cell culture was dark adapted for 30 minutes, the last 5 of which was recorded with the PAM sensor for F_0_ determination. Next, red (659 nm, half width 16.3 nm) light was used which increased in intensity every two minutes. The first two light intensities used were the growth-light intensity of the different strains; i.e. 30 and 60 μmol photons m^-2^ s^-1^. Next the light intensity was increased to 100 μmol photons m^-2^ s^-1^ and further increased in steps of 50 μmol photons m^-2^ s^-1^ to a final intensity of 400 μmol photons m^-2^ s^-1^. Two minutes before the actinic light was switched on and one minute after each change in light intensity, a strong light pulse was given (white light, 2,000 μmol photons m^-2^ s^-1^). This pulse is comprised of the different actinic LED channels set to maximal intensity; 800 μmol photons m^-2^ s^-1^ at 659 nm, 800 μmol photons m^-2^ s^-1^ at 620 nm and 400 μmol photons m^-2^ s^-1^ at 447 nm. After the light period the cells were returned to darkness for 5 minutes. Two minutes after onset of the dark period, samples for OD_680_/OD_730_ measurements were taken. Following the recommendation of Campbell et al. [19] the light was switched on again at 400 μmol photons m^-2^ s^-1^ and DCMU was added at a final concentration of 20 μM, and the DCMU response was followed for 7 minutes. From the obtained traces the values for F_0_ in the dark after dark adaptation, F_0_' in the dark after illumination, F_M_ after a strong white light pulse in the dark and F_M_ after addition of DCMU were determined. With the F_0_ and the highest F_M_ values variable fluorescence; F_V_ = F_M_-F_0_, the quantum yield of PSII; ϕ_PSII_ = F_V_/F_M_, photochemical quenching; q_P_ = (F_M_'-F)/ (F_M_'-F_0_') and non-photochemical quenching; q_N_ = 1-((F_M_'-F_0_')/ (F_M_-F_0_)) were calculated [25,26].

### Chl *a* content

For an estimate of the biomass-specific chl *a* content, the optical density of the culture was determined at 680 and 730 nm. The chl *a* content was then derived from the difference between OD_680_ and OD_730_ on the basis of a baseline of extracted chlorophyll in 80% (v/v) acetone/5% (v/v) DMSO (*Synechocystis*) or 90% (v/v) methanol/0.1% (w/v) magnesium bicarbonate (*Chlorella*). The chl *a* concentration in acetone was calculated as 12.7*OD_665_ (*Synechocystis* does not contain chl b, so OD_645_ was not included in the equation), and chl *a* concentration in methanol was calculated as (13.9*OD_665_)-(2.16*OD_645_).

### Determination of the photon requirement of biomass formation and of electron pair generation for carbon and nitrogen incorporation into biomass

All strains used were grown in a 2L flat panel chemostat [42] in BG-11 with 10 mM Na_2_CO_3_, the culture was bubbled with air + 1% CO_2_ at a rate of 10 L h^-1^ for *Synechocystis* and 60 L h^-1^ for *Chlorella*. The culture was kept at 30°C and illuminated with LED light boards (Philips) set at an output intensity of 105 μmol photons m^-2^ s^-1^ of red (659 nm, half width 16.3 nm) light for *Synechocystis* WT and *Chlorella* or 75 + 30 μmol photons m^-2^ s^-1^ of red (659 nm) and blue (447 nm, half width 15.3 nm) light for *Synechocystis* PAL. The dilution rate was set at 0.071 h^-1^ and 0.070 h^-1^ for *Synechocystis* WT and *Chlorella* respectively and 0.023 h^-1^ for *Synechocystis* PAL. In the steady state the light passing through the culture (I_out_) was measured at 10 different spots on the chemostat vessel and samples for OD_730_ and dry weight (DW) measurement were taken. The light available to the culture (I_in_) was measured at the same 10 spots, as the amount of light passing through the chemostat vessel containing only medium. The photosynthetic efficiency was then calculated as mol photons absorbed per gram biomass formed or as photons absorbed/electron pairs generated. To arrive at these numbers we used the I_in_ and I_out_ values to calculate how much light was absorbed in 1 cm2 in 1 hour and we used the growth rate and dry weight values to calculate how much biomass was produced in this square centimeter times the 5 cm depth of the culture for the actual volume in one hour. To convert biomass to the amount of CO_2_ fixed and nitrate assimilated, we averaged the carbon/nitrogen contents of 49.38/10.74% [43], 51.38/11.29% [44] and 49.8/12.5% [45] for *Synechocystis* and 47.54/6.73% [46], 42.54/6.64% [47] and 52.8/5.7% [48] for *Chlorella*. To arrive at the amount of electron pairs generated per gram of biomass formed, we used an electron requirement of 8 electrons per nitrate and 4 electrons per CO_2_.

### MIMS measurements

Membrane-inlet mass spectrometry (MIMS) makes use of two stable oxygen isotopes ^16^O and ^18^O, to measure oxygen release (via splitting of H_2_^16^O at PSII) and uptake of ^36^O_2_ by respiration, respectively. Abundance of these two oxygen isotopes is then analyzed via high-vacuum-supported diffusive equilibration through a gas permeable membrane, coupled to a mass spectrometer [41,75]. MIMS measurements were performed in a 10 ml air-tight cuvette containing a *Synechocystis* culture, with a density of 2 mg L^-1^ chlorophyll *a*. The high-vacuum membrane inlet sensor of the mass spectrometry analyzer was placed in the liquid culture. A thin Teflon membrane secured continuous passage of small amounts of gasses out of the liquid phase into the sensor tube of the mass spectrometer. Prior to the experiment, the sample was dark adapted for 30 minutes and then briefly (~ 10 sec) sparged with N_2_ to reduce the prevalent O_2_ concentration to about 20% of the value in air-equilibrated incubation buffer, with the aim to prevent O_2_ saturation during the experiment. After sparging, the cuvette was closed and ^36^O_2_ was added in the head space which, while stirring, equilibrated with the liquid. An up-sloping mass spectrometer signal denoted the ^36^O_2_ gas entering the solution until a plateau was reached at about 1ppm. The cultures were subjected to increasing 659 nm light intensities ranging from 10 to 400 μmol photons m^-2^ s^-1^. The lowest light intensity at the start of the experiment was on for 10 minutes to secure light adaptation and all subsequent light intensities were on for 3 minutes. After the light incubation, dark respiration was followed for 10 minutes.

## Supporting Information

S1 Fig Schematic representation of the set-up for PAM fluorimetry measurements. Schematic representation of the PAM fluorimetry set-up. 1, air supply (10 L h^-1^ air + 1% CO_2_); 2, hollow glass rod; 3, flat panel tissue culture flask (Sarstedt); 4, syringe for sampling; 5, PAM fluorimeter with a glass fiber rod for excitation/detection pressed to the side of the flask; 6, stir bean; 7, LED panels for actinic illumination and strong light pulses.
(EPS)
